# A scoping review of the evidence for community-based dementia palliative care services and their related service activities

**DOI:** 10.1186/s12904-022-00922-7

**Published:** 2022-03-09

**Authors:** Niamh O’Connor, Siobhan Fox, W George Kernohan, Jonathan Drennan, Suzanne Guerin, Aileen Murphy, Suzanne Timmons

**Affiliations:** 1 Centre for Gerontology and Rehabilitation, School of Medicine, University College Cork, Cork, Ireland; 2 Centre for Gerontology and Rehabilitation, The Bungalow, St Finbarr’s Hospital, Block 13, Douglas road, T12XH60 Cork, Republic of Ireland; 3 Institute of Nursing and Health Research, Ulster University, Newtownabbey, Northern Ireland; 4 School of Nursing and Midwifery, University College Cork, Cork, Ireland; 5 School of Psychology, University College Dublin, Dublin, Ireland; 6 Department of Economics, University College Cork, Cork, Ireland

**Keywords:** Dementia, Alzheimer’s disease, Palliative care, Community care

## Abstract

**Background:**

Palliative care is identified internationally as a priority for efficacious dementia care. Research into “effective models” of palliative care for people with dementia has been recommended by several European countries. To build an effective service-delivery model we must gain an understanding of existing models used in similar settings. The study aim is to identify core components of extant models of palliative care for people with dementia, and their families, who are living at home in the community.

**Methods:**

A scoping review was employed. The search strategy was devised to identify all peer-reviewed research papers relating to the above aim. This process was iterative, and the search strategy was refined as evidence emerged and was reviewed. All types of study designs and both quantitative and qualitative studies of non-pharmacological interventions were considered for inclusion.

**Results:**

The search identified 2,754 unique citations, of which 18 papers were deemed eligible for inclusion. Although a palliative care approach is recommended from early in the disease process, most evidence involves end-of-life care or advanced dementia and pertains to residential care. The majority of the research reviewed focused on the effects of advance care planning, and end-of-life care; specialist palliative care input, and/or generalist palliative care provided by dementia services to enable people to remain at home and to reduce costs of care. Community staff training in palliative care appeared to improve engagement with Specialist Palliative Care teams. Integration of dementia and palliative care services was found to improve care received for people with dementia and their carers.

**Conclusions:**

While the evidence for integration of dementia and palliative care services is promising, further high-quality research is necessary particularly to identify the key components of palliative care for people living with dementia. This is imperative to enable people with dementia to inform their own care, to stay living at home for as long as possible, and, where appropriate, to die at home.

**Supplementary Information:**

The online version contains supplementary material available at 10.1186/s12904-022-00922-7.

## Background

As a progressive, incurable disorder, dementia is amenable to a palliative care approach, from the point of diagnosis [1]. However, although people with dementia (PwD) have multiple palliative care needs [2], they are less likely to receive specialist palliative care (SPC) compared to people with cancer [3]. Only a minority of European Countries have sufficiently available palliative care services for PwD [4]. Compounding the problem, the number of people requiring palliative care in the UK may increase by up to 42.4% by 2040, with dementia the main driver; comparable rises are expected in other European countries [5].

Addressing this deficit is a European-wide policy objective [6]. Dementia palliative care is identified as a research priority by patient and public groups [7]; other expert groups have called specifically for research into effective care models [8, 9]. The EAPC White paper provides a framework for palliative care for PwD [10] highlighting the following research priority areas: person-centred care, communication and shared decision-making; optimal treatment of symptoms and providing comfort; and setting care goals and advance planning.

Hospital and hospice services are under immense pressure from [11] the rapidly rising rates of dementia globally, thus integrating and boosting palliative care through integrated community-based care models is a particularly important part of the response. Particular challenges face community care models, e.g. services may be organised differently based on geography and availability of services may be inequitable and PwD living at home may be less likely to be diagnosed and to be under a service [11]. The pressure on healthcare budgets may also be alleviated by adopting community-based models of dementia care, which are associated with increased quality-of-life, but at half the cost, compared to residential care models [12]. An understanding of the economic value of community dementia palliative care interventions is crucial to support their development.

A targeted survey of experts in the UK and Ireland identified key components specifically for a community-based dementia palliative care model, including carer support; continuity of care; interventions to support meaningful living; care planning and advance care planning; information, education and training [11]. To establish the evidence for these and other components of dementia palliative care, we undertook a scoping review. Most PwD want to stay living in the community, including at end-of-﻿life [13]; however existing research has focussed on residential care settings. Furthermore, while palliative care involves comprehensive symptom assessment and management from diagnosis, previous reviews have focused on pharmacological care and on advanced disease or end-of-life [14]. The current aim is to uncover evidence on the efficacy of non-pharmacological components of community dementia palliative care.

## Method

 A scoping review was chosen as the most appropriate review method, as our aim was to map out evidence, e.g. the evidence type available and knowledge gaps, rather than to produce synthesized conclusions regarding effectiveness [15]. This suited the expected diversity in study dementia stage and terminology, and the expectation that some palliative care activities might not be defined as such by study authors. Furthermore, we anticipated little published high-quality quantitative research. We adhered to the steps outlined by Levac et al. [16]; to ensure a rigorous and transparent process.

The 5-stage framework includes: identifying the research questions; defining eligibility criteria; study selection and search strategy refinement; data charting; collating, summarising, and reporting results. The scoping review protocol was registered with Prospero, ID: CRD42018091158.

### Identifying the research questions

The scoping review method facilitated an iterative but rigorous process in developing the research questions. We initially considered all non-acute settings for dementia palliative care, however early searches uncovered existing research and reviews in residential care settings, thus we focused on uncovering the evidence for dementia palliative care for PwD living in the community. We were initially highly inclusive of study types and examined simple service descriptions, however, our searches revealed sufficient papers containing effectiveness data. The type of effectiveness data reported varied widely, thus a scoping review and summary was upheld as the appropriate choice, over a systematic review (SR) and meta-analysis.

Our final research questions were:

1. What is the evidence for the effectiveness of non-pharmacological components of community dementia palliative care, provided individually or together?
2. What evidence exists regarding the cost or cost-effectiveness of dementia palliative care interventions?

### Defining eligibility criteria and study selection

The databases MEDLINE, EMBASE, CINAHL, Scopus, and PsychInfo were searched for articles published 01/Jan/1995-01/March/2020 (supplementary file 1). The search was managed using “*Covidence”* online review software. Titles and abstracts were first screened using the broad original inclusion criteria by one of three researchers (SF, NOC or AR). Full-text candidate papers were independently screened by two researchers who met to discuss conflicts and reach resolution (NOC, SF or ST). The reference lists of the included studies were screened; no further studies were located. The wider research team regularly reviewed and refined the search strategy; resulting in the revised research questions stated above and two corresponding changes to the inclusion criteria. The original and revised inclusion criteria are detailed in Table 1. We included studies including participants with moderate-advance dementia; most studies clearly stated this, otherwise ST (geriatrician with clinical dementia expertise) reviewed the population details to confirm dementia stage. We were inclusive of broad definitions of palliative care, including papers where the author identified the intervention as “palliative care” or where the intervention corresponded to a specified component such as advance care planning (ACP), goal setting, carer bereavement support, etc. [10].

**Table 1:** Original inclusion criteria and revisions made during the scoping review process:

Original criteria	Revised criteria	Reason for change
***Inclusion criteria***		
PwD, or carers of PwD, living in the community in their own family home or a nursing home (NH) setting.	PwD or carers of PwD living in the community in their own family home.	Sufficient studies were found in community setting outside of NH settings.
People with moderate to advanced dementia, of any type.	No change.	
Studies including any type of data on dementia palliative care services or interventions (i.e. including descriptions of services).	Studies which reported impact or effectiveness data.	Sufficient studies were found which provided effectiveness data.
Papers reporting quantitative and/or qualitative data.	No change.	
Papers published between 1st of Jan 1995 and March 2020.	No change.	
Published peer reviewed papers in English.	No change.	
***Exclusion criteria***		
Studies based in acute hospitals.	No change.	

### Charting the data and collating, summarising and reporting results

A data-charting form was developed for data extraction (using Microsoft Excel). As mixed data types were included, the quality appraisal followed the Hawker framework [17]. This was conducted by two reviewers (NOC and SF/EOS); with discrepancies resolved by a third reviewer (ST). No papers were excluded based on the quality appraisal.

When summarising the results, the evidence is presented first for individual interventions/components, followed by overall service models. Where a study contained evidence for several components, findings are presented under the predominant component. Components identified were: ACP, education for family members and carers, education and training for staff, spirituality and therapeutic activities, bereavement support for families/carers; Service models identified were: dementia services providing palliative care for PwD, SPC-provided services for PwD, and integration/linking of existing services. We use SPC instead of ‘hospice’ care (which specifically denotes in-patient palliative care in some countries). We also differentiate SPC from a more generalist palliative care approach that may be adopted by community and dementia services, whereas SPC implies a multidisciplinary team (MDT), led by a palliative care consultant, who only provide palliative care. Exact outcome measures and effect sizes are presented for trial data, but not for quasi-experimental data.

## Results

### Search results

**Fig. 1:**
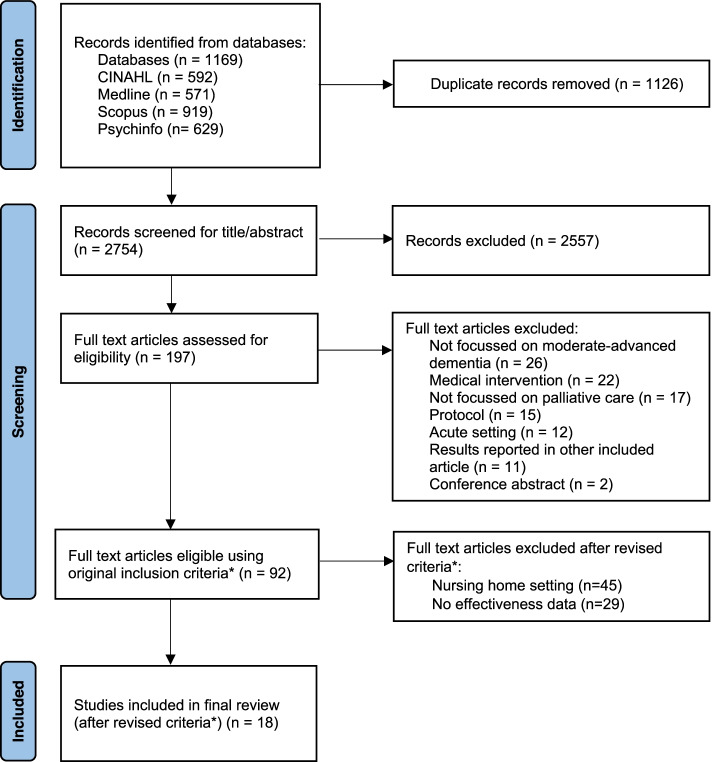
Prisma flow diagram. *In line with the scoping review process, we refined the study inclusion/exclusion criteria during study screening phase

Initially 3880 papers were identified through database searches; 18 papers were included in the final review (Fig. 1).

### Characteristics of included studies

Details of included studies are in Table 2. Regarding methodologies, there were 4 SRs which met our inclusion criteria. Of all the original studies, including those reported within a SR, the study designs were: Randomised control trials (RCTs) (*n *=12); retrospective cohort (*n *=6); prospective cohort (*n *=3); cross-sectional (*n *=4); pre-post (*n *=3); case-control (*n *=2) studies. Regarding location, the original studies were conducted in the US (*n *=11), the UK (4), Australia (*n *=4), Italy (*n *=2), Singapore (*n *=2), and Netherlands, France, Russia, Israel, Japan, Hong Kong, Peru (each *n *=1).

**Table 2:** Details of Included Studies

Author (year)	Study Design	Origin/ Country	Aim/Purpose	Population/ Sample size	Intervention type i.e. component under review	Outcome measurements	Key findings
Tay et al., (2020) [50]	Prospective cohort	Singapore	The objective of this study was to identify modifiable factors associated with the comfort of dementia patients dying at home and families’ satisfaction with care.	202 deceased people with dementia, with a median age of 88.	A palliative home care programme consists of a skilled MDT, e.g. trained ACP and in the use of dementia-specific tools to assess patient’s symptoms and QoL.	Relevant outcomes included the Comfort Assessment in Dying with Dementia scale which assessed dying patients’ comfort. The Satisfaction with Care at the End-of-Life in Dementia scale evaluated family caregivers’ satisfaction 2 months after bereavement.	Independent factors associated with families’ satisfaction with care were comfort [β (95% CI) =0.149 (0.012-0.286), *P *=.033] and honouring of medical intervention preferences (96.0%) [β (95% CI)=3.969 (1.485-6.453), *P *=.002].
Tilburgs et al., (2020) [24]	RCT- single blinded cluster	Netherlands	To explore the effects of an educational intervention for GPs.	38 Dutch GPs across different practices. 140 PwD across the selected practices.	Intervention group GPs were trained in ACP, including shared decision-making and role-playing exercises. Control group GPs provided usual care. at 6 month follow-up participants medical records were analysed using random effect logistic and linear regression.	Primary outcome was ACP initiation. Secondary outcomes included the number of medical and non-medical preferences discussed with the GP.	Compared to the ‘care as usual’ GPs, the GPs in the intervention group discussed a mean additional 0.8 medical preferences (95% CI: 0.3, 1.3; *p *=.003), and 1.5 nonmedical preferences (95% CI: 0.8, 2.3; *p* <.001) per PwD.
Bryant et al., (2019) [22]	SR (relevant included study = Pre/Post test)	Mixed, (1 relevant = US)	To examine the effectiveness of interventions in increasing ACP for PwD.	4 studies, 1 relevant	Advanced Care Treatment-Plan intervention with caregivers of PwD involving weekly educational sessions across 4 weeks delivered by advanced practice nurse facilitators. Intervention took place in adult day care centres.	Knowledge about dementia and EoL treatments, Self-efficacy regarding ACP and decisions to develop an ACP for PwD.	Findings strongly suggest that the ACT-Plan intervention is feasible and appropriate for caregivers. The intervention group showed an increase in outcomes under study compared to the control group. Those in the intervention group also developed more ACP.
Hum et al., (2019) [49]	Prospective cohort study	Singapore	To explore patients’ symptoms and quality-of-life, and their association with eternal feeding. To evaluate the impact of the programme on these parameters and examine family caregiver burden.	458 patients were enrolled in the homecare service, of which 254 were included in the analysis of this study	Homecare service with an MDT of doctors, nurses, and medical social workers who make regular home visits and phone calls during office hours. During visits the homecare team assessed patients’ needs to manage pain and behavioural symptom. Caregivers were also given education on a range of topics.	Pain (Pain Assessment in Advanced Dementia). Patient symptoms (Neuropsychiatric Inventory Questionnaire). Quality of life (Quality of Life in Late-Stage Dementia). Family caregiver burden (ZBI).	This integrated multidisciplinary palliative homecare team which is trained and accessible at all hours addressed the needs of people with advanced dementia living in the home, improved the QoL, and supported families to care for their loved on at home.
Jennings et al., (2019) [53]	Retrospective cohort	US	To evaluate the effect of a dementia care co-management model on subsequent end-of-life care	PwD treated by the service who later died (N-322). Medical records of last 6 months looked at retrospectively. .	The model involved dementia service nurse practitioners partnering with primary care providers and community organisations to provide comprehensive dementia care to PwD (any stage or severity).	Place of death. Discussions of future care.	The majority had a SPC discussion or consultation in their last 6 months of life, 69% had SPC input at the time of death, and 66% died at home. PwD with an advance healthcare directive within the service were more likely to have had a discussion about SPC (78% vs. 64%; *P* = .01), die with SPC input (74% vs. 62%; *P* = .03), and die at home (70% vs. 59%; *P* = .04).
Miranda et al., (2019) [15]	SR (8 relevant: RCT=5; case-control=2; cross-sectional=1)	Mixed (8 relevant: US=4, Italy=2, UK=1, Japan=1)	To examine evidence on home palliative care interventions in dementia in terms of effectiveness on EoL care outcomes.	Eight studies were included in the review, all home based.	A range of home based palliative care interventions involving specialist and non-specialist palliative care interventions.	A range of outcomes including patient death at home, institutionalisation, functional status, behavioural symptoms at EoL, pain, satisfaction and resource use.	The interventions which commonly focused on optimal symptom management, continuity of care and psychosocial support, showed the potential benefits of the interventions in improving EoL care outcomes.
Moore et al., (2019) [27]	SR (5 relevant: RCT=5)	Mixed (5 relevant = US, Hong Kong, France, Russia, Peru)	To explore whether interventions incorporating education regarding the progressive nature of dementia increased carers’ understanding of dementia and improved mental health burden.	11 studies were included, 5 were relevant	All were multicomponent interventions which included education/information on progressions of dementia as a core component.	Burden (Zarit Burden Interview/ Family Caregiving Burden Inventory). Knowledge of dementia (self reported on a visual analogue scale). Depression (Beck Depression Inventory).	There was no significant evidence found to support or disprove the effectiveness of education on progression of dementia on carers knowledge and mental health.
Sternberg et al., (2019) [51]	Pre/post quality improvement study	Israel	To examine clinical and health services outcomes of a quality improvement pilot project to provide home hospice care for older people with advanced dementia.	People with advanced dementia (*N *=20) being treated in the Maccabi Healthcare Services homecare program,	Study participants received home hospice care as an extension of their usual care for 6–7 months (or until they died) by an MDT who were available 24/7. The MDT included nurses, occupational therapists, social workers, spiritual care. MDT members took part in workshops to improve communication between teams and enhance dementia knowledge.	Family members were interviewed using validated questionnaires, i.e. Satisfaction with care (Volicer validated tools).Caregiver burden (Zarit Burden Interview) scale. Advanced dementia prognostic tool (ADEPT). Functional and cognitive status (FAST tool). Hospitalizations prevented and medications discontinued, were determined by medical record review and team consensus.	Results suggest that home hospice care for people with advanced dementia can improve symptom management and caregiver satisfaction, while decreasing caregiver burden, preventing hospitalisations and discontinuing unnecessary medications.
Dixon et at,. (2018) [19]	SR (2 relevant: retrospective cohort = 1; cross-sectional=1)	Mixed, (2 relevant = US)	To review existing evidence concerning the effectiveness of ACP in improving EoL outcomes for PwD.	18 studies total, 3 relevant	ACP interventions, preserving identity, planning for ACP, treatment limiting advance directive & written advance directive	Depression (CSDD), QoL (BASQID), Coping (IMMEL), Self reported anxiety (CSDD), Medicare spending, hospital death, ICU use, place of death, comfort in dying (CAD-EOLD)	Having ACP and advanced directives in place can lead to a number of improvements for PwD and carer, however, there is a need for more randomised designs.
Harrison et al., (2018) [55]	Prospective cohort study (pilot)	UK	This paper describes and discusses an innovative partnership between Dementia UK and a UK hospice, and outcomes from year one evaluation and sets out future plans.	50 referrals were received between December 2016 and September 2017 (*N *=39 PwD, *N *=11 carer).	Pilot study introducing the first EoL care Admiral Nurse to a Specialist Community Palliative Care Team to identify and support PwD who required palliative and EoL care.	Level of functionality (Barthel Index). Satisfaction with end-of-life care (SCW-EOLD). Preferred place of death. Inappropriate hospital admissions.	Of the 12 deaths within the Admiral Nurse caseload during year 1, most PwD (*n *=10) died in their usual place of residence i.e. community, and preferred place of death.
Harrop et al., (2018) [55]	Cross sectional	UK	A pilot study to introduce the first EoL care Admiral Nurse to specialist community palliative care team to identify and support PwD who require palliative and EoL care.	Total of 35 surveys were completed (20 HSCP, 9 current carers, 6 bereaved carers).	An innovative service for palliative care of EoL care for PwD introduced into a UK hospice which assessed the addition of an Admiral Nurse to the team.	Number of referrals to hospice palliative care team. Number of referrals of PwD being care for in their home. Family satisfaction	75% increase in referrals of PwD to the hospice palliative care team. 287.5% increase in referrals of PwD being care for in home since the pre-project year (*n *=8) and first year of the project (*n *=31). 98% of carers rated the service extremely helpful (*n *=10) or quite helpful (*n *=4). 69% of carers indicated an improvement in their knowledge, confidence and practical skills (*n *=11).
Spilsbury et al., (2017) [48]	Retrospective cohort study	Australia	To determine if community-based palliative care was associated with reduced hospital costs in the last year of life, and to compare people dying from caner and those from other conditions.	Cohort of all who died between Jan 2009 and Dec 2010 in Western Australia from life limiting condition including dementia.	A palliative nurse consultancy service was available to residential care facilities. The service included advise, assessment, staff education and telephone follow up. Client dates of enrolment and disenrollment were used to define periods of time receiving community-based palliative care.	Day-specific hospital costs: mean day-specific hospital cost as being a cohort average hospital cost, mean day specific hospital cost averaged over only decedents who were in hospital on a particular day.	Community based SPC was associated with hospital cost reductions across multiple life-limiting conditions including dementia.
Rosenwax et al., (2015) [46]	Retrospective cohort	Australia	To describe patterns in the use of hospital emergency departments in the last year of life by people who died with dementia and whether this was modified by use of community-based palliative care.	All people who died with dementia in Western Australia over 2 years (*N *=5261). A comparative cohort of people who died from other conditions amenable to palliative care (*N *=2685).	Looking retrospectively at the effect of PwD who received community based palliative care and the number of emergency department admissions in last year of life, versus those who did not receive it.	Hospital admissions	Only 6% of the dementia cohort used community-based palliative care compared to 26% of the other cohort. For PwD, the provision of community-based palliative care was associated with a significantly reduced daily rate of ED visits over the last year of life, with a six-fold difference at the EoL.
Toye et al., (2015) [26]	Pre/Post study	Australia	Action research study conducted to trial a strategy intended to support a consistent palliative approach for PwD nearing death.	130 eligible staff took part. 30 families took part. Note: Numbers differed for different parts of intervention (educational sessions, educational booklet etc.).	Two plans were focused on in this paper which were providing information and education to staff and supporting families. Educational sessions and information booklets were mainly used, with the intervention being informed by numerous experts.	Knowledge (Dementia Knowledge Assessment Tool, Palliative Approach Questionnaire). Wilcoxon Signed Rank Test comparing pre and post scores from staff educational session. View of practice change collected by ‘end of study’ interviews with family carers.	Staff support and information sessions and resources were rated positively by staff but the actual impact in on knowledge, views, and confidence were small. Family feedback for the sessions and resources ‘supporting families’ was primarily positive.
Chang et al., (2010) [25]	Cross sectional	Australia	To investigate the application of a new dementia information booklet for family caregivers in both the community and residential care.	672 information booklets were distributed to family carers (*n *=129 across the dementia services). 233 carers completed the evaluation questionnaire (not specified how many from community only).	The booklet, *Information for Families and Friends of People* *with Severe and End Stage Dementia* (Palliative Care Dementia Interface: Enhancing Community Capacity Project, 2006), formed the fundamental material of this project	Carer satisfaction with the booklet, Knowledge about dementia. Outcomes were measured by questionnaires and qualitative data gather from interviews.	72% indicated that their preference was to receive information either at the onset of dementia or at the time of the diagnosis of dementia, or soon after diagnosis. 97% of the carers found the booklet to be helpful and that the booklet contained sections that were useful (96%). 86% of carers felt it should be provided freely.
Treloar et al., (2009) [41]	Exploratory retrospective Cohort	UK	Detailed interviews of key carers who had supported PwD at home were carried out, with the aim of identifying major factors which make such care feasible.	Key carers interviewed more than three months after the death of PwD (*N *=50)	A novel service supported persons with advanced dementia to live and die at home. The service is run by a psychiatrist of old age and includes an advanced nurse practitioner acting as a key worker to coordinate care for the PwD, get necessary equipment, support the carer. Staff are available by phone out of hours.	Interviews were completed with carers, to discus satisfaction with care etc. Authors didn’t specify specific outcomes.	This study demonstrates that good, home based palliative care of dementia can be achieved with very positive outcomes. Bereavement may be helped by the process of caring at home till death. Key factors for success include the right equipment, expertise around relevant medication, food, and social care needs, as well as understanding and support for funding care commissioning and informal care.
Haley et al., (2008) [37]	RCT	US	To examine the joint effects of bereavement and caregiver intervention on caregiver depressive symptoms.	Primary carers (*N *=254) who cared for their spouse at home	Enhanced bereavement support which comprised of three components: two individual and four family counselling sessions, weekly support session for caregivers, and ‘ad hoc’ counselling available for duration of study.	Depressive symptoms and carer resilience (Geriatric Depression Scale)	Lower depressive symptoms were found in the intervention group compares to the control group both before and after bereavement.
Shega et al., (2008) [39]	Retrospective cohort	US	To evaluate the impact of hospice enrolment i.e. SPC, on the care of PwD and describe the symptom burden PwD experience.	Family members of persons who have died with dementia (*N *=135).	PwD enrolled to a hospice programme compared to non enrolees. The researchers were blinded to the patients hospice enrolment status.	Location of choice for PwD. Hospital deaths for PwD. Pain and burdensome treatments for PwD (Verbal Descriptor Scale). Caregiver satisfaction with care (interviews, pats adapted from the Toolkit of Instruments to Measure EoL Care).	PwD enrolled in hospice were significantly more likely to die in their location of choice, and less likely to die in hospital, compared to those who weren’t enrolled. Caregivers of enrolees were more satisfied with the care their loved one received than non-enrolees.

Where systematic reviews were described only the outcomes and findings of the relevant studies included within the review are discussed.

*Abbreviations*: *SR* Systematic Review, *RCT* Randomised Control Trial, *ACP* Advance Care Planning, *EoL* end-of-life, *GP* General Practitioner, *HSCP* Health and Social Care Professionals, *PwD* People with Dementia, *QoL* Quality of Life, *SPC* Specialist Palliative Care

Most studies were of moderate quality (Supplementary file 2). Out of maximum of 36 for the Hawker quality appraisal scale, the range of scores was 22-34. Across the studies, the subscales receiving the lowest quality scores overall were “implications and usefulness”, “transferability/generalisability”, and “ethics and bias”. However, the most “poor” and “very poor” individual appraisals were given for the categories concerning methods and data analysis.

### Advance care planning (ACP)

The initial search yielded considerable evidence relating to ACP, but few studies related to community-dwelling PwD.

Dixon et al [18] present a SR of 18 studies reporting the potential effectiveness or cost effectiveness of ACP for PwD and/or their carer, where two studies are relevant to this review.

A US population cohort study [19], combining health retirement survey data and health insurance provider data, included 3876 participants in total, with 2064 community-dwelling, and the remainder in nursing homes. The cohort included people with severe dementia (21.7%) in both settings. Within the cohort of community-dwelling people with severe dementia, those who had an advance healthcare directive (AHD) in place had lower levels of intensive care unit use (9.5%), reduced in-hospital death (18%) and 35% lower healthcare costs ($11,461 less costs in the last 6 months of life). This study was unable to identify the cost of performing the AHD.

A retrospective survey of 156 bereaved relatives in the US [20], where the PwD had died in the preceding year, and had spent all of their last 3 months of life in residential care (*N *=77), or at home (*N *=24), or had transitioned from home to residential care in this time (*N *=53). Pooled across the three groups, compared to those with an AHD, those without an AHD had 7.1 additional hospital days during the last year of life and 7% more in-hospital deaths, but no significant difference in caregiver satisfaction with care, or their perception of the PwD’s symptom control.

An SR by Bryant et al. [21] included one relevant study which explored the feasibility of implementing an ACP intervention with 68 caregivers of PwD [22]. During a four-week pre/post intervention study, the carers, enrolled from adult day care centres in African-American urban communities in Chicago, were assigned to the ACP intervention, or a control group who received general health promotion information. Caregivers rated the intervention as feasible and appropriate. Intervention group caregivers more often developed ACPs and had increased self-efficacy, and knowledge about dementia and end-of-life treatments.

A single-blinded cluster RCT by Tilburgs et al. [23] looked at the effect of training Dutch General Practitioners (GPs) in ACP. In total, 38 GP practices took part. PwD aged over 65 with any type of dementia were recruited (*N *=140). GPs in the intervention group were trained in ACP while control group practices provided usual care. After 6 months, intervention group GPs initiated ACPs with 35 PwD (49.3%), and control group GPs with 9 PwD (13.9%). The intervention group GPs also discussed a higher number of medical and non-medical preferences with PwD.

### Education for family members and carers

Four studies provided evidence to support providing information or education to families and carers.

Chang et al. [24] evaluated a dementia information booklet, distributed via 14 dementia advisory services to 672 family caregivers in Australia, of whom 129 were caregivers for a community-dwelling PwD. The booklet provided information about disease progression, behaviours and emotions, physical symptoms, issues surrounding end-of-life and palliative care options, preparing for death and the dying process, and signposting available supports. Of 223 caregivers who completed an evaluation questionnaire (26% response rate), including 33 responses from family caregivers of a community-dwelling PwD, 96% found the booklet useful, 86% felt it should be available free of charge, and 72% would want to receive this information at the point of diagnosis, or very soon after.

Toye et al. [25] used action research to facilitate a Community of Practise to support a palliative approach to care for people with advanced dementia across settings in Western Australia. For community-dwelling PwD, the intervention included in-home respite and counselling services provided by a dementia advocacy and support organisation and a home-care service provider. Health and social care professionals (HSCPs) also provided education and support for 28 families using an existing carer information booklet during a planned conversation with a staff member trained on ‘grief and loss in dementia’. This booklet contained information about dementia progression and encouraged consideration of issues such as the dying process. Additionally, a carer education session, developed with Community of Practice input, was delivered by a counsellor and medical practitioner to 22 families; a list of useful contacts was also provided to 30 families. Feedback obtained through user surveys (response rate 29%, *N *=12/41), was favourable that the intervention helped with future decision-making.

Moore et al. [26] conducted an SR exploring whether interventions, including education about dementia progression, increased carers’ understanding of dementia and subsequently improved mental health. In total, 11 studies were included, with five of these community-based, delivered in the person’s home [27–30], or as a day service in a local nursing home [31]. All were RCTs of multicomponent interventions, where one component aimed to increase carers’ understanding of dementia progression. Interventions were delivered using various formats such as group-based [31], home-based [29, 30], one-to-one [27] and a computerised intervention which could be accessed from home [28]. The interventions provided strategies for managing “behavioural and psychological symptoms of dementia” and improving communication and decision-marking skills; information on environmental safety, social, financial, and psychological support for carers; teaching carers to tailor everyday activities to the PwD’s ability. Outcomes included depression (Beck Depression Inventory), caregiver burden (Zarit Burden Interview) and self-reported knowledge of dementia. Overall, the results were mixed, with the SR authors noting that the quality of evidence for improved outcomes was low. Knowledge of dementia was evaluated in only one community-based study [28], where a statistically significant difference favouring the intervention group was found at 3 months but not at the 6-month assessment.

A further RCT, included in an SR of home-based palliative care interventions by Miranda et al. [14], involved training and supporting carers of community-dwelling people with moderate to severe dementia to use a comprehensive individualised person-centred management approach, with the aim of reducing “behavioural disturbances” [32]. The control group (*N *=10) received usual care. Intervention group carers (*N *=10) received 8 educational sessions that provided task-specific training and useful information on the course and personal impact of Alzheimer’s disease, and management of behavioural disturbances, nutrition and carer’s stress, followed by ten further fortnightly caregiver support sessions. Both groups received memantine at a therapeutic dose (to allow comparison to a previous memantine trial by the same group [33]). The primary outcome was the Clinicians Interview-Based Impression of Change Plus Caregiver Input (CIBIC-Plus), which rates cognition, function, behaviour and activities of daily living. A 3-point (clinically significant) benefit was found in the intervention group versus the control group throughout the 28-week study duration.

### Education and training for staff

Staff education was often a key element of multicomponent interventions, but its effects were rarely reported separately. Two relevant studies were found.

Toye et al. [25] included staff education as part of their action research Community of Practise intervention, discussed earlier. Some 60 HSCPs across home-care services completed baseline surveys, with 15% identifying educational needs related to end-of-life communication skills, pain assessment, management of terminal delirium, and end-of-life ethics. Education was then provided to 114 of 130 eligible staff, such as nurses, care managers and allied health professionals (86%); no GPs were included. Overall, the staff education and informational resources were evaluated positively by recipients. In the 74 individuals who completed before-after training surveys, median dementia knowledge scores increased slightly but significantly.

Another study reported in the Miranda et al. [14] SR involved education of a SPC team to provide palliative care to community-dwelling PwD. Nakanishi et al. [34] conducted a cluster-randomised controlled trial with HSCPs who provided home care to PwD in Japan, comparing the Behaviour Analytics & Support Enhancement (BASE) programme to usual care. The programme consisted of a 2-day staff training course which helped HSCPs to identify PwD’s unmet needs, an online tool to aid HSCPs in monitoring for and assessing symptoms, and a multi-agency team meeting. Intervention group HSCPs (*N *=49) provided four to six home visits per week across several weeks, according to individual needs. The control group home care providers (*N *=49) did not receive the training or tool and provided usual care. HSCPs performed the Neuropsychiatry Index in the PwD receiving care from the intervention group (*N *=141) and control group (*N *=142) at baseline and follow-up. There was a clinically significant reduction in total Neuropsychiatry Index total scores at 6-month follow-up in the intervention group (mean reduction 7 points), with minimal change in the control group.

### Spirituality and therapeutic activities

Our review found studies concerning spirituality published only from 2015 onwards (although none met our criteria for inclusion) suggesting that spirituality is an emerging research area in dementia palliative care. Regarding therapeutic activities, only one paper within the review by Miranda et al. [14] was community-based [35]. Multi-sensory stimulation (non-directed, with non-sequential stimuli across all senses, and no physical or cognitive demands) was compared to control activity sessions (non-multisensory, staff-directed, and usually sequential stimuli) within an RCT (intervention group *N *=25; control group *N *=25). The participants were home-bound with moderate to severe dementia; sessions were delivered at two day centres by trained therapists. Those who received Multi-sensory stimulation demonstrated significant improvement in mood and behaviour (Behaviour and Mood Disturbance Scale mean difference -3.72 (CI: -7.1, -0.34, *p *=.032)) and in social disturbance (Behaviour Rating Scale) mean difference -0.84 (CI: -1.59, -0.09, *p *=.029)), versus the control group whose behaviour progressively declined. When adjusted for pre-trial differences, scores changed slightly for the Behaviour and Mood Disturbance Scale (*p *=.051), and the Behaviour Rating Scale (*p *=.074). Furthermore, behaviour deteriorated in the intervention group during the follow-up period after sessions were stopped.

### Bereavement support for families/carers

One relevant study investigated bereavement support for families. An RCT, by Haley et al. [36], examined the effects of a bereavement support intervention on carers’ depressive symptoms. All participants were primary carers for their spouse at home. The control group (*N *=132) received usual care. The intervention group (*N *=122) received enhanced bereavement support as follows: two individual and four family counselling sessions, weekly carer support sessions, and additional counselling as needed during the two-year study. Depressive symptoms (Geriatric Depression Scale) decreased among carers in both groups following the death of the PwD, with the largest decrease seen where the PwD had not transitioned into residential care. Control group carers were more than twice as likely to be chronically depressed than intervention group carers (intervention group=7.8%, control group=17.9%, *P *=.0439), with resilience also significantly higher in the intervention group (intervention group=60%, control group=42.9%, *P *=.0234). In addition, there was a beneficial impact from early carer support (i.e. not just when end-of-life was anticipated), with improved mean levels and patterns of depressive symptoms (i.e. the 8 bereavement patterns identified by [37]) while the PwD was alive, and post-death.

### Dementia services providing palliative care for PwD

Moving from individual components to service models, our search revealed six studies of dementia services providing generalist palliative care to community-dwelling PwD.

Shega et al. [38] used retrospective surveys of family members of a deceased PwD to evaluate the impact of in-home palliative care. The Palliative Excellence in Alzheimer Care Efforts (PEACE) research programme [39] was coordinated by primary-care based geriatric services in Chicago, with nurse specialists liaising between families and the MDT. The programme incorporated ACP, patient-centred care, family support, and a palliative care approach from diagnosis until end-of-life. PwD enrolled in the research programme (*N *=58) were more likely to die in their location of choice and less likely to die in hospital than those not enrolled (*N *=77). Enrolled carers were more likely to rate care as ‘very good’ or ‘excellent’. There was no improvement in symptom frequency or severity, or distress or pain.

Treloar et al. [40] collected feedback from carers about a UK psychiatry community service which supported people with advanced dementia in their homes, including support for dying at home. Interviews with family carers of 14 PwD who availed of the service indicated that critical success factors included having the right equipment (hoists), gaining knowledge around needs and services, and providing carer support. The authors, using the assumption that all 14 PwD would otherwise have required nursing home care for the year prior to death, estimated that the cost saving for that year was substantial (£696,930, 2009 figures).

Four studies in the SR by [14] aimed to deliver hospital-type care in the PwD’s home. One study evaluated medical care provided by house calls [41] by a physician-nurse practitioner team, the latter also supporting patients as they transitioned to/from hospital and attended follow-up hospital clinic appointments. PwD in the study (*N *=144) were more likely to have expenditures related to home health and hospice (and not hospital or residential care) than PwD not receiving this care (matched health insurance participants, *N *=440). This expenditure pattern suggests reduced hospitalisations and residential care.

Two papers reported on the results of an RCT in Italy, involving a Home Hospital Service, which focused on bringing all critical elements of hospital care to the home setting of acutely ill patients, including PwD, who had been admitted to hospital and were eligible for discharge home with this service [42, 43] (intervention group *N *=56; control group *N *=53). The Home Hospital Service functioned daily from 8am to 8pm, delivered by geriatricians, nurses, physiotherapists, and a social worker, counsellor and dietitian. Communication with the caregiver was identified as key, including discussing the care plan and encouraging them to phone with any questions or issues. Intervention group PwD had significantly lower rates of transition to residential care (28.5% decrease) and a higher percentage of dying at home (31.4% more). Caregiver stress was also reduced with the Home Hospital Service support.

Another study [44], included in the Miranda et al. [14] review, evaluated the PATCH (Palliative Access Through Care at Home) programme which involved geriatrician-led MDTs improving palliative care for older adults living at home, of which the majority were PwD (64%, *N *=75). Specific interventions included environmental assessment, communication around goals of care, ensuring availability of care, and medical interventions. Many of the PwD died at home or in a hospice (35.7% and 28.6% respectively); one-third died in hospital (35.7%) and no-one in residential care. Furthermore, most carers and PwD were extremely satisfied or very satisfied with the intervention.

### SPC-provided services for PwD

A number of studies reported evidence for SPC-led services for PwD.

Rosenwax et al. [45] conducted a retrospective population-based cohort study of PwD (*N *=5216) who died in 2009/2010 in Western Australia, compared to a random sample within an age and sex-matched cohort without dementia who died from conditions known to require palliative care during the same period (*N *=2685). In this region, >90% of home-based SPC is provided by a not-for-profit group, which provides home care and in-home SPC, via an MDT, in predominantly urban areas [46]. The service includes physical care, practical support, symptom management, counselling and bereavement support, ACP, respite, and links to other services. Within the PwD cohort, only 6% received this community-based SPC, compared to 26% of the comparator cohort. In a subsequent study, the research group [47] compared hospital care costs in the final year of life between those receiving the service or not in a sample of 12,764 people with cancer and non-cancer conditions who died in 2009/2010, of whom 605 were people with Alzheimer’s disease (AD). Reductions in hospital costs were again seen in the AD cohort receiving the service, across the final year of life.

A prospective cohort study [48] evaluated a homecare service provided by a palliative homecare team with geriatric training to people with advanced dementia (*N *=254) in Singapore. The MDT were available ‘as needed’ for issues which could not be resolved via telephone support. Carers were educated on non-pharmacological interventions, environmental modifications, and music therapy to relieve discomfort. Psychological and emotional support was provided to carers, and the MDT worked closely with the PwD’s hospital physician to avoid the emergency department (ED), if appropriate, including direct admission to inpatient hospice care if needed. The PwD’s neuropsychiatric symptoms, nutritional status, pain and quality-of-life (stratified by mode of feeding: enteral versus oral) improved significantly from baseline to month five of the service (*N *=54). Caregivers were stratified based on living arrangements and availability of stay-in help (not provided as part of the intervention). Availability of stay-in help was found to significantly reduce caregiver burden median scores for all burden factors except for ‘inadequacy’.

A second paper by this group [49] focused on modifiable factors associated with the comfort of PwD dying in their homes (Comfort Assessment in Dying with Dementia scale), and families’ satisfaction with care, as rated two months post bereavement (*N *=202). Approximately 80% of PwD died at home. Family satisfaction with care was independently influenced by honouring medical intervention preferences and by perceived comfort at end-of-life.

Sternberg et al. [50] conducted a quality improvement project examining clinical and health service outcomes for 20 older people with advanced dementia in Israel, who received SPC-provided home care for 6-7 months, or until death. Following staff workshops to improve team communication, and knowledge of dementia, swallowing problems, hand feeding and managing “behavioural problems”, the intervention involved scheduled monthly visits from a physician and weekly visits from a nurse, with 24/7 availability of telephone support, and extra home visits as required. Social workers conducted an initial assessment and contacted PwD by a home visit or telephone call every 2 weeks. Spiritual workers were involved where necessary. The visits and interactions were more frequent than usual care, where physicians visited every 3-6 months, nurses every 1-3 months and social workers 1-2 times per year. The intervention improved symptom management (Volicer symptom management scale), and family satisfaction with care and caregiver burden. There were only five hospitalisations during the programme, with an additional 33 hospitalisations ‘prevented’, and a mean of 2.1 medications discontinued per participant.

Miranda et al’s [14] SR included an observational retrospective case-control study which assessed a hospital-to-home transition programme developed to improve palliative care for home-bound individuals with chronic conditions including dementia [51]. This programme included training for SPC MDTs to provide in-home interventions including medical consultations, ACP, caregiver support, education for carers regarding illness trajectory and treatment choices, and spiritual support. Outcome data pertained to resource use in the transition-support group (*N *=92), versus a propensity-matched control group (*N *=276). The transition-support group had lower hospital, non-hospital and total healthcare costs per month. This group also had fewer deaths in hospital, fewer hospital days per month and 25.7% less admissions to intensive care unit in the last month of life.

### Integration/linking of existing services within the community

Three papers concerned partnerships across services in the community setting.

Jennings et al. [52] conducted an observational cohort study of a dementia care co-management model on subsequent end-of-life care. The model involved dementia service nurse practitioners partnering with primary care providers and community organisations in Los Angeles to provide comprehensive dementia care (any stage or severity). In total, 322 PwD who had been treated by the service and later died (ranging from 1 to 44 months of service receipt) had retrospective electronic medical health record data collected for their last 6 months of life. Over half had no hospitalisations or ED visits and the majority had a SPC discussion or consultation in this time; 69% had SPC input at the time of death; and 66% died at home. Those who had completed an AHD within the service were more likely to die with SPC input, and die at home.

There were positive effects in two separate UK-based pilots where SPC and dementia teams were integrated [53, 54]. Harrop et al. [53] describe a partnership between a hospice and the Alzheimer’s Society, delivered through a community SPC nurse specialist and a dementia support worker. The support includes regular visits and telephone calls, which may involve patient care, information, advice, education, and training. There was a 75% increase in the first year of the project in referrals to the SPC team compared to the pre-project year, and a nearly 300% increase in referrals of community-dwelling PwD. Family carers reported increased knowledge, confidence, and practical skills across a range of palliative care issues. HSCPs rated the project as ‘extremely helpful’ (*N *=13) or ‘quite helpful’ (*N *=1).

The other pilot [54] involved an Admiral Nurse (specialist dementia nurse) joining the community SPC team to provide dementia expertise for people with advanced dementia, with current or closely anticipated ‘unresolved, complex needs that cannot be met by their current care team’. An important service component was the opportunity for ACP discussions. Reflecting this, of the 12 deaths within the Admiral Nurse caseload during the first year, ten died in their usual place of residence, noting that pre-service data for comparison is not available.

## Discussion

This review found evidence to support several components of dementia palliative care, including ACP, education for family members and carers, education and training for staff, spirituality and therapeutic activities, and bereavement support for families/carers. However, many studies identified included complex interventions or service activities where the effect of individual components could not be isolated. Several service models have some evidence for their value, including SPC-delivered, geriatric/dementia service-delivered and integrated joint services and partnerships. Overall, there was a dearth of high-quality RCTs or reasonably sized pre-post studies, such that there is no clear best-practice approach or model for providing palliative care to community-dwelling PwD and their families. Large, longitudinal studies would be required to fully demonstrate outcomes across the life-course of dementia, requiring significant resources. However, smaller, quasi-experimental studies, such as were included in the current review, often reflect real-world rather than trial situations, hence providing valuable insights for service provision. Economic evidence across the studies was sparse. Some studies reported on resource utilisation costs, but no full economic evaluations assessing cost-effectiveness were identified. However, the limited evidence would suggest a trend towards the potential cost-effectiveness of dementia palliative care interventions, primarily through reduced intensive care and hospital admissions, shorter length of stays, and avoidance of residential care. Further high-quality economic evidence, assessing both intervention costs and valuation of benefits is needed to support the development of new models.

A further challenge is that dementia palliative care in the literature often relates to advanced dementia and end-of-life. Current National Institute for Health and Care Excellence (NICE) guidance supports a palliative approach from diagnosis until end-of-life for PwD [55]. Furthermore, as discussed by [25], a palliative approach which is targeted and incremental, with interventions over a longer time period, is required to address the needs of PwD. The Irish National Dementia Strategy [56] is in a minority among national policies [57] in highlighting in its palliative care section the need for timely dementia diagnosis and disclosure, to anticipate and plan for cognitive/communication loss, and to support ACP that involves the PwD. It also supports the ethos that palliative care should begin from diagnosis/disclosure and continue until end-of-life, and beyond as bereavement support for families, reflecting newer thinking on palliative care [58].

Our review highlights the feasibility of facilitating ACP discussions with community-dwelling PwD, through primary care [23, 59], and with family caregivers of a community-dwelling PwD [22], noting that wherever possible, ACP should involve the PwD, not just their family. The United Nations Convention on the Rights of Persons with Disabilities [60] supports the rights of a person with a disability, which includes PwD, to make their own decisions. As reinforced by Robinson et al. [61], waiting until advanced stages of dementia and/or residential care for ACP is often too late; these conversations need to be held at an earlier stage to allow the PwD to make decisions and communicate their wishes and values. There is a significant literature base on ACP in residential care settings, as a common place of death for many PwD and where it is easier to conduct large scale studies [61]. In many of these studies, the family makes the decisions *for* the PwD rather than *with* them; in other retrospective studies, the process around the previously decided AHD or ACP is not presented. There is a clear requirement for community-based services for PwD to incorporate ACP as a core element of service provision, as a natural extension of everyday conversations around disease progression and symptoms, and of getting to know the person and what is important to them. It’s imperative that staff are properly trained to recognise triggers to undertake timely ACP, and have the confidence and time to support PwD in this process [62].

 Our review included several studies that explored education and information for family caregivers. The caregivers valued materials such as information booklets, but there was mixed evidence for education or information improving carer’s dementia knowledge, mood or caregiver burden. Education on person-centred care improved global function in one small RCT [32].

Spirituality is a key pillar of palliative care, but we found no studies which addressed this for community-dwelling PwD [36]. We found limited evidence for therapeutic activities as part of palliative care for community-dwelling PwD. Only one RCT addressed this, with positive outcomes for multisensory stimulation in terms of mood and ‘behaviour’ [35].

We were particularly interested in overall service models. Studies presenting evidence for different models came from several different countries representing varied contexts, lending a broad perspective to our results. Our review found evidence for SPC teams upskilling in dementia care, for dementia services providing palliative care, and for service integration. While using different models, services shared similar components, such as: link/key worker staff roles (linking with families and across teams); staff training; specialist staff coming to the person’s home; ACP; practical support, physical care, environmental assessment, and equipment (e.g. hoists); medical consultations and symptom management; counselling, bereavement support, spiritual support; respite, caregiver support, caregiver education; and communication with the caregiver, especially around goals of care. It is essential that carer support and a dyadic approach is central to any model which aims to support PwD to live at home [63].

Advocating for a palliative approach to all care for PwD could help supplement usual dementia care with specific palliative care components, wherein primary/community and dementia care services would adopt “generalist palliative care” approaches, incorporating ACP, proactive symptom prediction and assessment, and bereavement support in their usual dementia care. Staff training could help to alleviate any residual lack of understanding and awareness around a palliative care approach in dementia care. Equally, SPC services providing a service to PwD has growing evidence, and training SPC staff in dementia care is feasible. Issues here may relate more to service capacity for people with non-malignant diseases.

Some components of optimal palliative care for PwD, as identified in the EAPC white paper [10], were missing or poorly represented in this review, including continuity of care, and person-centred care. These are part of usual dementia care, with evidence for these available in multiple other reviews and studies (e.g. [64, 65]). In addition, as we did not look at specific medical interventions, symptom management and providing comfort was, by design, under-represented in our included studies.

In terms of the optimal model of dementia palliative care for a given country, intuitively there should be integration between SPC, primary/community care and dementia-specific services, with tailored and flexible input within and between services according to the PwD’s current need [66, 67]. However, in parallel, we must address wider system issues such as dementia under-diagnosis hindering access to services, delayed diagnosis/disclosure denying the PwD their right to make decisions about their current and future care, and residual stigma around palliative care. Thus, adequate diagnostic services and pathways and public information campaigns around palliative care are a crucial pre-requisite for any successful model for dementia palliative care.

The current evidence supports that community-based dementia palliative care is feasible to deliver. There are many studies evidencing a palliative care approach at end-of-life in nursing homes; introducing care in these settings is facilitated by onsite medical and nursing staff, equipment, 24/7 care, etc. However palliative care interventions in such settings may focus on pharmacological interventions, or those addressing artificial nutrition and hydration, etc. [68, 69]. People with dementia living at home have better quality-of-life, compared to their counterparts in residential care [70], and a truly holistic and person-centred model of palliative care may be more feasible in home settings.

Undertaking a review of community dementia palliative care is inherently complex. Community support is a broad construct in itself. “Effectiveness” of a palliative care approach is complex as a balance must be struck between maintaining autonomy and the idea of the “self” while accepting that the trajectory of dementia will ultimately result in a loss of these, and planning for this eventuality. As mentioned earlier, general palliative care overlaps greatly with standard dementia care. While we endeavoured to keep our inclusion and exclusion criteria broad, it was necessary to detail them to a level to allow for a systematic search and summary. Thus, the complexity of community dementia palliative care may be limited. This challenge is inherent to the topic, and is something of which researchers, policy-makers, and others assessing evidence in this area should be aware.

### Limitations

Grant and Booth [71] imply that a lack of quality assessment limits the uptake of scoping review findings into policy and practice. We mitigated this by reviewing the quality of the included papers. However, given the subject area, the evidence was generally of moderate quality. Some relevant studies may not have used the term ‘palliative care’ in their title or abstract, and so did not appear in our search, although we attempted to mitigate this by using a broad search strategy. Grey literature was not searched, so relevant reports or unpublished work may not omitted. Medical interventions were outside the review scope, so we did not look at evidence around optimal symptom treatment or clinical decision-making. While we set out to include cost-effectiveness evidence for dementia palliative care interventions, little evidence existed.

## Conclusions

 This review has examined evidence-based non-pharmacological interventions for community-dwelling PwD. There is emerging evidence to support community-based, dementia palliative care services, and their common service components. Specifically, evidence was found for the components of: ACP, education for family members and carers, education and training for staff, spirituality and therapeutic activities, and bereavement support for families/carers. Our review highlights the need for further interventional studies, implementation studies and evaluations of dementia palliative care services for community-dwelling PwD, to enable PwD to inform their own care, to continue to live at home for as long as possible, and, where appropriate, to die at home.

## Supplementary Information

**Additional file 1.** List of search terms used in search strategy.

**Additional file 2.** Quality appraisal of included studies using the framework of Hawker et al. (2002).

## Data Availability

All data generated or analysed during this study are included in this published article and its supplementary information files.

## Abbreviations

ACP: Advance care planning
AD: Alzheimer’s disease
AHD: Advance healthcare directive
EAPC: European association for palliative care
ED: Emergency department
GP: General practitioner
HSCPs: Health and social care professionals
MDT: Multidisciplinary team
PwD: People with dementia
RCT: Randomised controlled trial
SPC: Specialist palliative care
SR: Systematic review
