# Supplementary material for: A scoping review of the evidence for community-based dementia palliative care services and their related service activities
Source: BMC Palliat Care. 2022 Mar 9;21:32. doi: 10.1186/s12904-022-00922-7 (PMC8905782; doi:10.1186/s12904-022-00922-7)
Supplement: Supplementary file 1 — Additional file 1. List of search terms used in search strategy. [file 12904_2022_922_MOESM1_ESM.docx]

**Supplementary file 1:** List of search terms used in search strategy

| **Database searched** | **Search terms used** |
| --- | --- |
| *Medline via Ebsco* | (MH "Dementia+") OR AB ( (dementia) or (Alzheimer*) ) OR TI ( (dementia) or (Alzheimer*) )  AND  ( (MH "Palliative Care") OR (MH "Hospice and Palliative Care Nursing") OR (MH "Palliative Medicine") ) OR AB ( (palliative) or (end of life care) or (comfort care) or (Symptom control) or (supportive care) or (end stage) ) OR TI ( (palliative) or (end of life care) or (comfort care) or (Symptom control) or (supportive care) or (end stage) )  AND  AB ( (model) or (framework) or (intervention) or (service) or (evaluation) or (pathway) or (programme) ) OR TI ( (model) or (framework) or (intervention) or (service) or (evaluation) or (pathway) or (programme) )  Limited to human, English and years 1995-2020. |
| *Embase* | 'dementia'/exp OR dementia:ab,ti OR alzheimer*:ab,ti  AND  model:ab,ti OR framework:ab,ti OR intervention:ab,ti OR service:ab,ti OR evaluation:ab,ti OR pathway:ab,ti OR programme:ab,ti  AND  'palliative therapy'/exp OR palliative:ab,ti OR 'end of life care':ab,ti OR 'comfort care':ab,ti OR 'symptom control':ab,ti OR 'supportive care':ab,ti OR 'end stage':ab,ti  Limited to human English and years 1995-2020 |
| *Cinahl via Ebsco* | (MH "Dementia+") OR TI ( (dementia) or (Alzheimer*) ) OR AB ( (dementia) or (Alzheimer*) )  AND  ( (MH "Hospice and Palliative Nursing") OR (MH "Palliative Care") OR (MH "Hospice and Palliative Nurses Association") ) OR TI ( (palliative) or (end of life care) or (comfort care) or (Symptom control) or (supportive care) or (end stage) ) OR AB ( (palliative) or (end of life care) or (comfort care) or (Symptom control) or (supportive care) or (end stage) )  AND  TI ( (model) or (framework) or (intervention) or (service) or (evaluation) or (pathway) or (programme) ) OR AB ( (model) or (framework) or (intervention) or (service) or (evaluation) or (pathway) or (programme) )  Limited to human English and years 1995-2020 |
| *Scopus* | TITLE ( dementia  OR  alzheimer* )  OR  ABS ( dementia  OR  alzheimer* ) ) )  AND  ( ( TITLE ( ( palliative )  OR  ( "end of life care" )  OR  ( "comfort care" )  OR  ( "symptom control" )  OR  ( "supportive care" )  OR  ( "end stage" ) )  OR  ABS ( ( palliative )  OR  ( "end of life care" )  OR  ( "comfort care" )  OR  ( "symptom control" )  OR  ( "supportive care" ) OR (“end stage”) ) OR ABS ( (palliative) IR (“end of life care”) OR (“comfort care”) OR (“symptom control”) OR (“supportive care”) OR (“end stage”)) AND ( (TITLE ( (model) OR (framework) OR (intervention) OR (service) OR (evaluation) OR (pathway) OR (programme) OR ABS ( (model) OR (framework) OR (intervention) OR (service) OR (evaluation) OR (pathway) OR (programme) ) )  Limited to human English and years 1995-2020 |
| *PsychInfo* | ( (DE "Dementia" OR DE "AIDS Dementia Complex" OR DE "Dementia with Lewy Bodies" OR DE "Presenile Dementia" OR DE "Semantic Dementia" OR DE "Senile Dementia" OR DE "Vascular Dementia") OR (DE "Alzheimer's Disease") ) OR TX ( (dementia) or (Alzheimer*) ) OR AB ( (dementia) or (Alzheimer*) )  AND  DE "Palliative Care" OR TI ( (palliative) or (end of life care) or (comfort care) or (Symptom control) or (supportive care) or (end stage) ) OR AB ( (palliative) or (end of life care) or (comfort care) or (Symptom control) or (supportive care) or (end stage) )  AND  TI ( (model) or (framework) or (intervention) or (service) or (evaluation) or (pathway) or (programme) ) OR AB ( (model) or (framework) or (intervention) or (service) or (evaluation) or (pathway) or (programme) )  Limited to human English and years 1995-2020 |
