# Supplementary material for: A scoping review of the evidence for community-based dementia palliative care services and their related service activities
Source: BMC Palliat Care. 2022 Mar 9;21:32. doi: 10.1186/s12904-022-00922-7 (PMC8905782; doi:10.1186/s12904-022-00922-7)
Supplement: Supplementary file 2 — Additional file 2. Quality appraisal of included studies using the framework of Hawker et al. (2002). [file 12904_2022_922_MOESM2_ESM.docx]

**Supplementary file 2:** Quality appraisal of included studies using the framework of Hawker et al. (2002)

| Study Authors | Abstract & title | Introduction & aims | Method & data | Sampling | Data analysis | Ethics & bias | Findings/ results | Transferability/ generalizability | Implications and usefulness | Score / 36 |
| --- | --- | --- | --- | --- | --- | --- | --- | --- | --- | --- |
| *Tay et al., 2020* | G | G | F | F | G | F | G | F | F | **31** |
| *Tilburgs et al., 2020* | G | P | G | F | G | F | G | F | F | **30** |
| *Bryant et al., 2019* | G | G | G | G | VP | G | G | F | F | **31** |
| *Hum et al., 2019* | G | G | G | F | G | P | G | F | F | **31** |
| *Jennings et al., 2019* | G | G | G | F | G | F | G | F | F | **32** |
| *Miranda et al., 2019* | G | G | G | G | G | G | G | F | F | **34** |
| *Moore et al., 2019* | G | G | G | G | F | G | G | F | F | **33** |
| *Sternberg et al., 2019* | G | G | G | G | VP | P | G | F | F | **29** |
| *Dixon et al., 2018* | G | G | G | G | F | F | G | F | F | **32** |
| *Harrison et al., 2018* | P | F | P | F | F | P | P | P | F | **22** |
| *Harrop et al., 2018* | F | G | P | VP | F | F | G | F | F | **26** |
| *Spilsbury et al., 2017* | G | G | G | G | G | G | G | F | F | **34** |
| *Rosenwax et al., 2015* | G | G | G | F | G | F | F | F | F | **31** |
| *Toye et al., 2015* | G | G | P | P | P | G | G | G | F | **29** |
| *Chang et al., 2010* | G | G | G | G | G | G | F | F | F | **33** |
| *Treloar et al., 2009* | F | G | F | G | F | VP | G | G | F | **27** |
| *Haley et al., 2008* | F | F | G | F | G | F | F | F | F | **29** |
| *Shega et al., 2008* | F | G | G | G | G | F | G | F | F | **34** |
| Index: Good (G)= 4 points Fair (F)= 3 points Poor (P)= 2 points Very Poor (VP)= 1 point  *Note: the quality appraisal was conducted independently by 2 reviewers and the total score represents the average of the two scores, where applicable. | | | | | | | | | | |
